# Supplementary material for: Imaging the dynamic influence of functional groups on metal-organic frameworks
Source: Nat Commun. 2023 Aug 10;14:4835. doi: 10.1038/s41467-023-40590-6 (PMC10415300; doi:10.1038/s41467-023-40590-6)
Supplement: Supplementary file 1 — Supplementary Information [file 41467_2023_40590_MOESM1_ESM.pdf]

Supplementary Information

Imaging the Dynamic Influence of Functional Groups on Metal Organic Frameworks

Boyang et al.

* Corresponding author:

Xiao Chen, chenx123@tsinghua.edu.cn;

Tiefeng Wang, wangtf@tsinghua.edu.cn

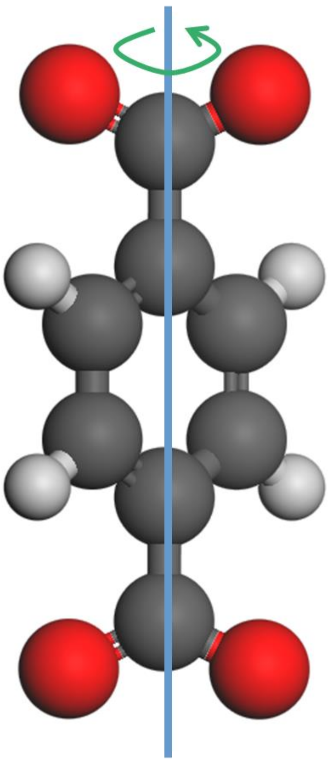

Supplementary Figure 1. Schematic model of π-flipping in BDC linkers.

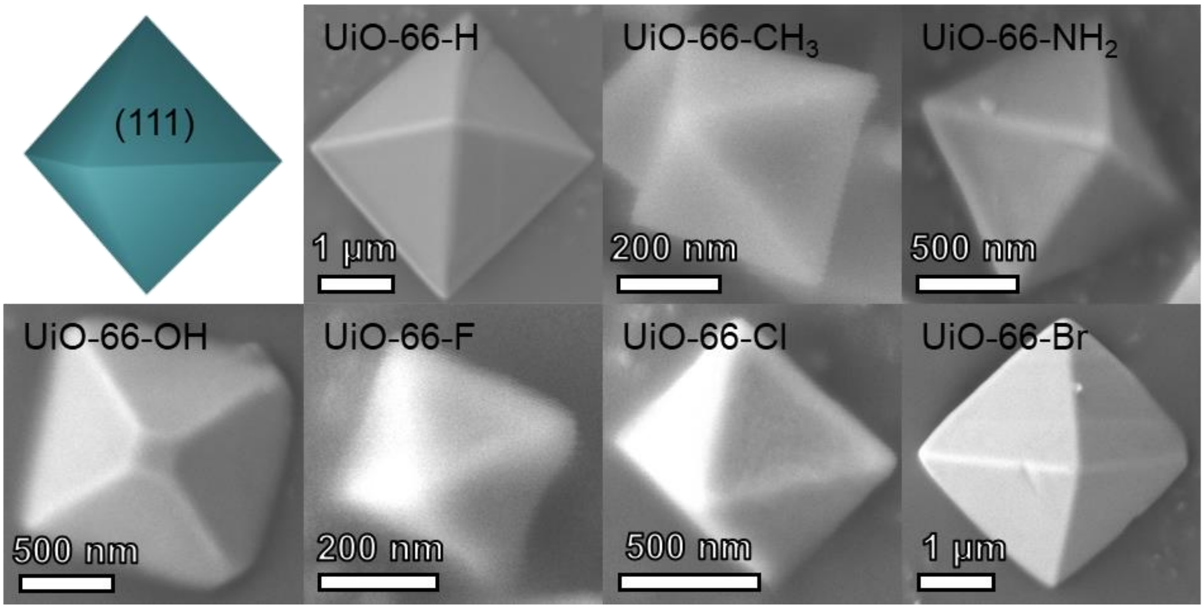

Supplementary Figure 2. Morphology of UiO-66-X. The SEM images of UiO-66-X showed octahedral morphology.

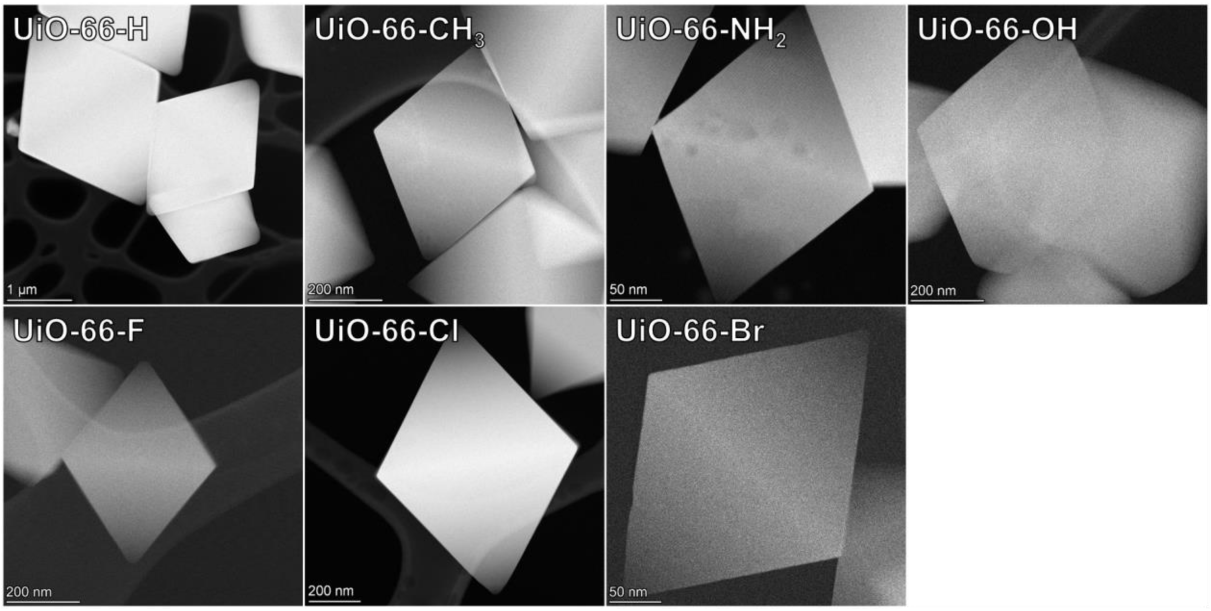

Supplementary Figure 3. HAADF-STEM images of UiO-66-X.

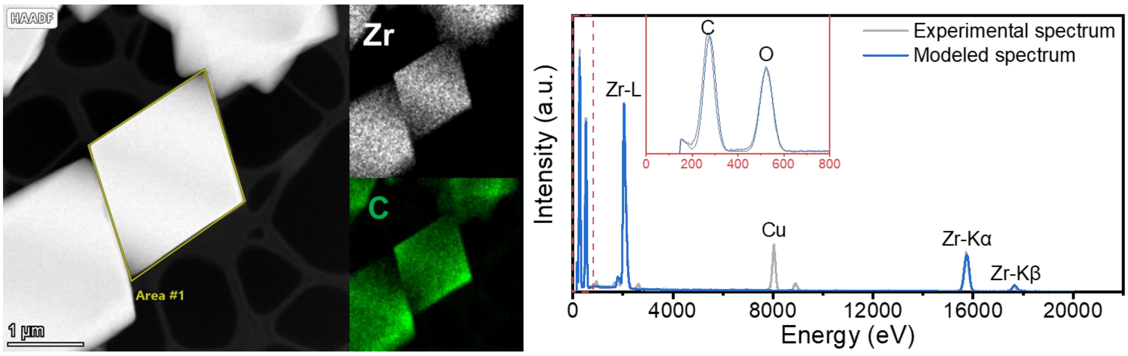

Supplementary Figure 4. EDS mapping of UiO-66-H.

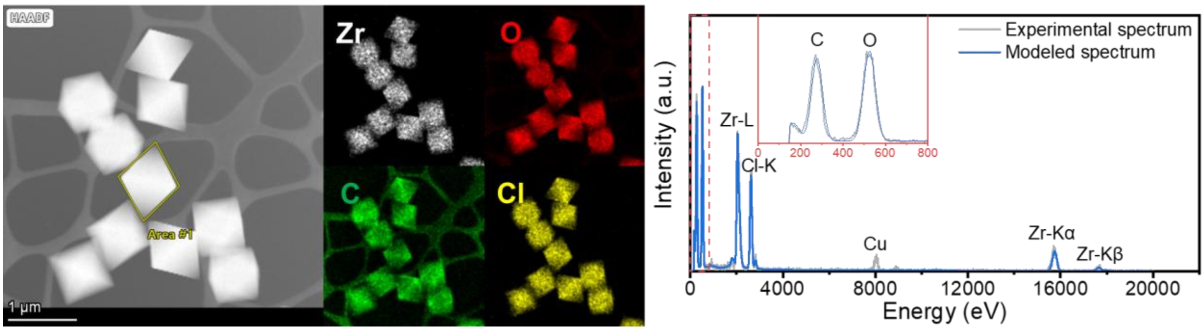

Supplementary Figure 5. EDS mapping of UiO-66-Cl.

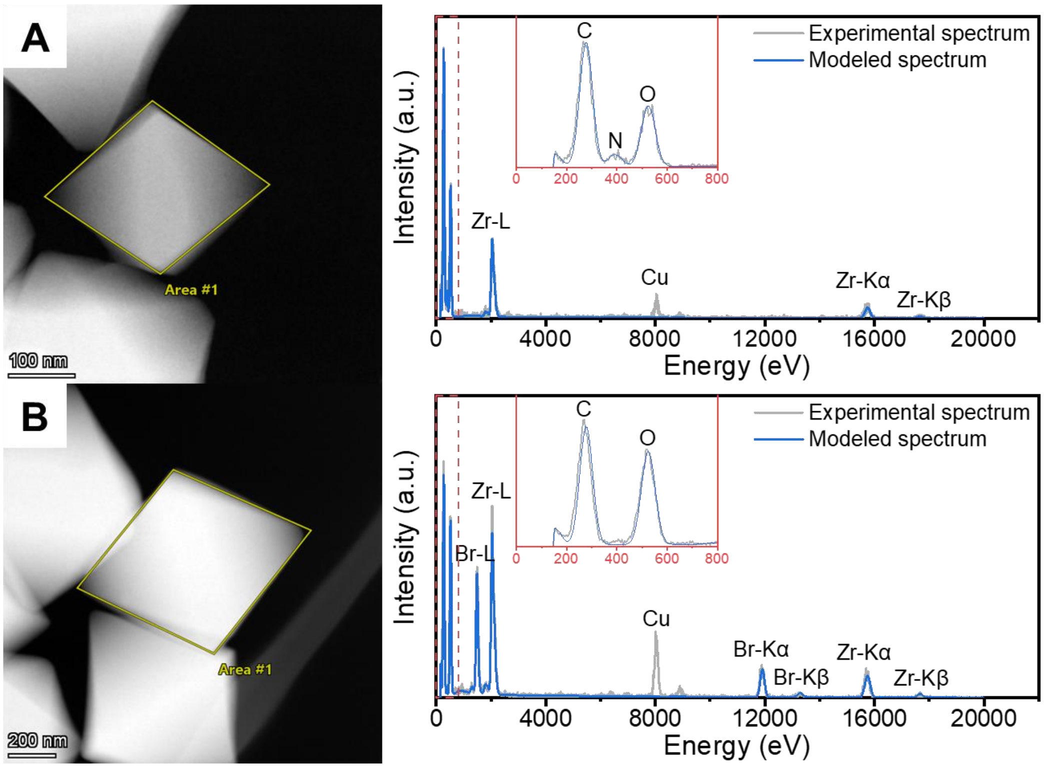

Supplementary Figure 6. EDS mapping of UiO-66-X. EDS spectrum of selected areas for UiO-66-NH_2 (A) and UiO-66-Br (B).

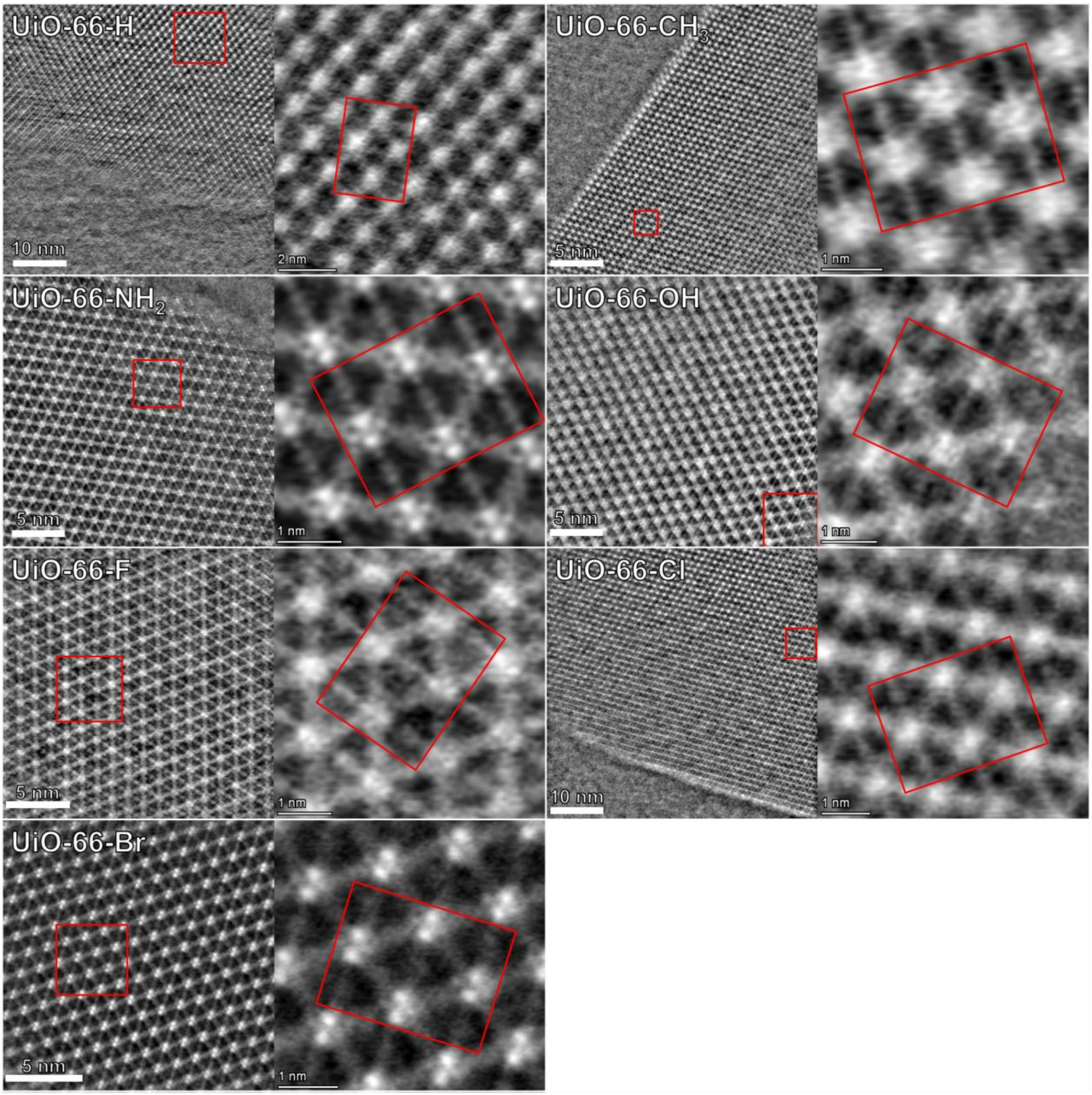

Supplementary Figure 7. Selected regions of iDPC-STEM images in Figures 2 and 3. iDPC-STEM images of UiO-66-X with low and high magnification. The selected areas are marked by the red rectangle.

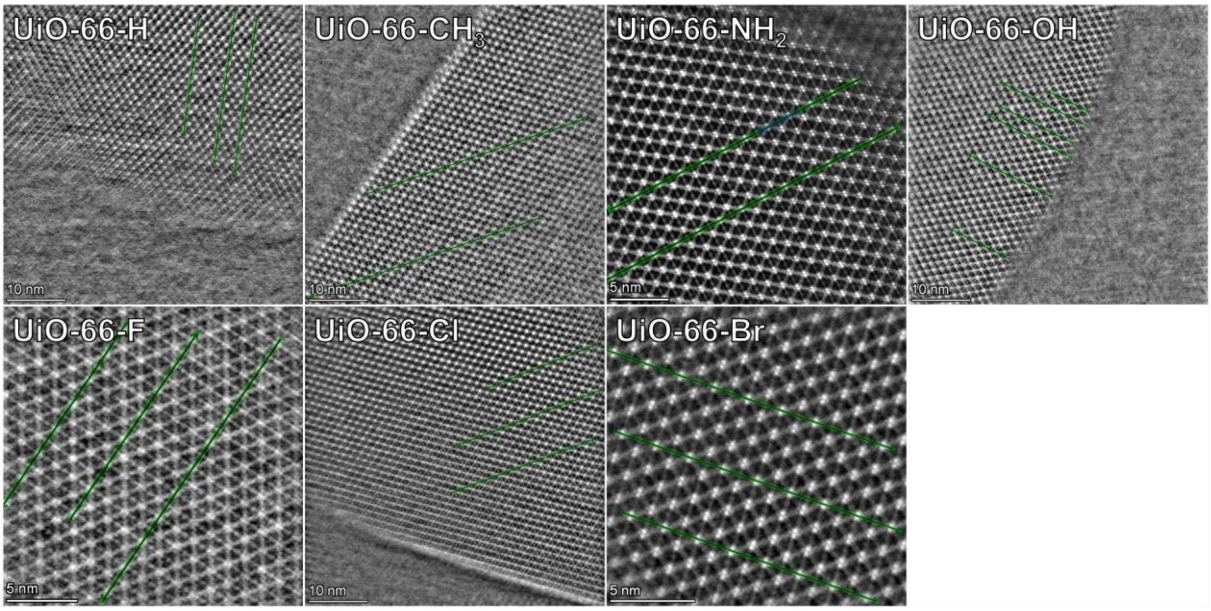

Supplementary Figure 8. Selected intensity profiles to obtain average FWHM. The intensity profiles along the green arrows are summarized to calculate the average FWHM in Figure 3F.

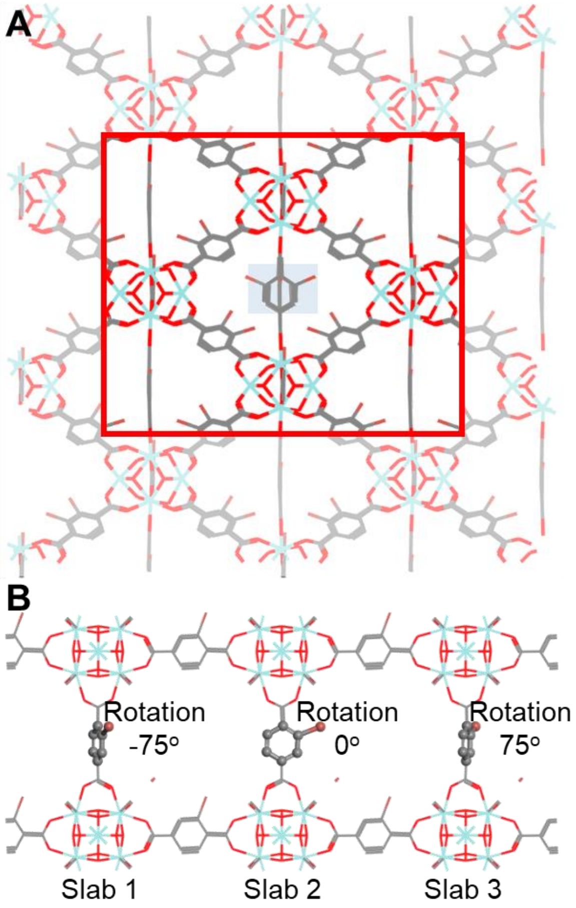

Supplementary Figure 9. Typical model of UiO-66-Br for iDPC-STEM simulations. (**A**) Top view of the three-layered model for simulation. The displayed region in Figures 2 and 3 is marked by the red square. The region highlighted by blue is attributed to the rotated BDC-Br linkers. The BDC-Br linkers in other cells remain unchanged. (**B**) Side view of rotated BDC-Br linkers. For simulation, we rotate the linker in the first slab by -75° and the linker in the third slab by 75°, while the BDC-Br linker in the second slab remains unchanged. The rotation degree of ±75° is calculated using 0.18 eV as the thermal motion energy. We use this model to simulate the distribution of different BDC-Br rotation angles. The grey, red, white and brown atoms represent C, O, H and Br elements, respectively.

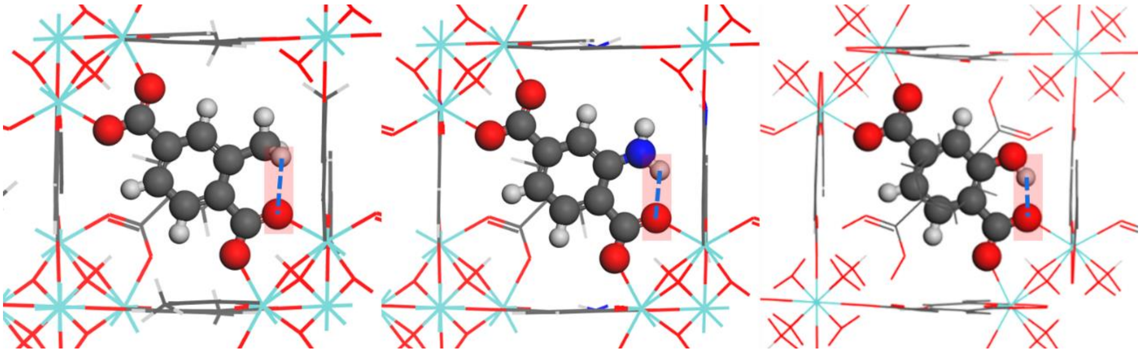

Supplementary Figure 10. Schematic diagram of intramolecular hydrogen bond in UiO-66-X. The intramolecular hydrogen bonds are highlighted in red and the H…O atoms are connected by dashed blue lines. The grey, red, white and blue atoms represent C, O, H and N elements, respectively.

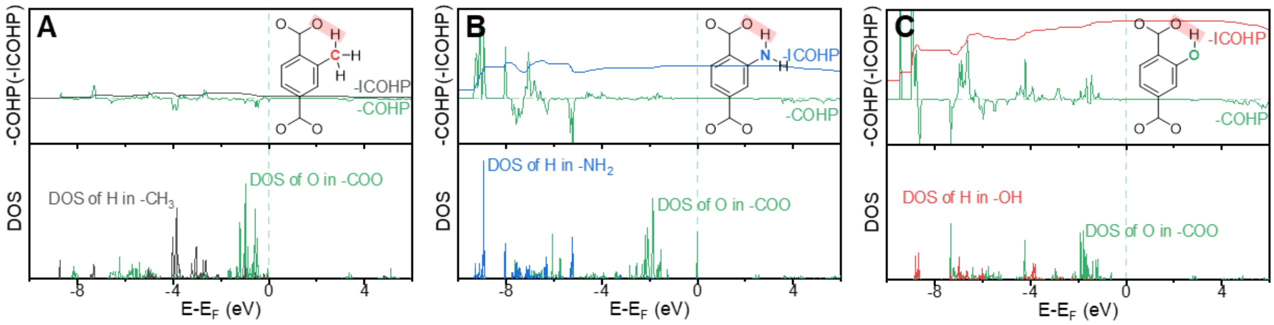

Supplementary Figure 11. Electronic structure of hydrogen bond. The density of states (DOS) are shown below. The DOS of adsorbed H is multiplied by 5 times. The overlap of DOS confirmed the existence of hydrogen bonds between O in carboxyl groups (-COO) and H in functional groups (-CH_3, -NH_2, -OH). The crystal orbital Hamilton population (COHP) and the integrated COHP (ICOHP) represent the bonding effects between O in carboxyl groups and H in functional groups. The calculated atom pairs are highlighted in the inset. The positive value of –COHP represent the bonding effect, while the negative value of –COHP represent the anti-boding effect.

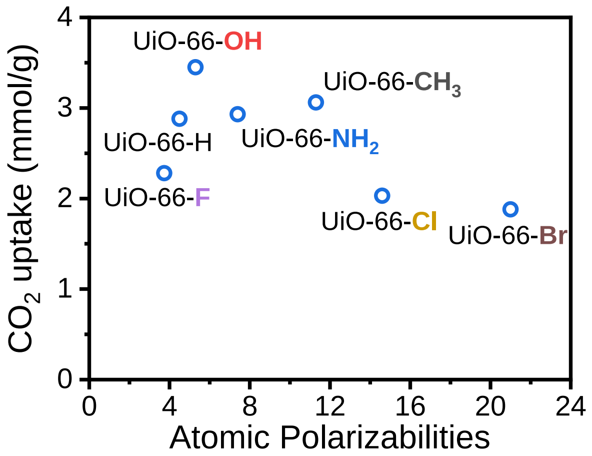

Supplementary Figure 12. Correlation between CO_2 uptake and polarizabilities of functional atoms. The polarizabilities are obtained from Ref.[50] and the values of CO_2 uptake are obtained from Ref.[13]

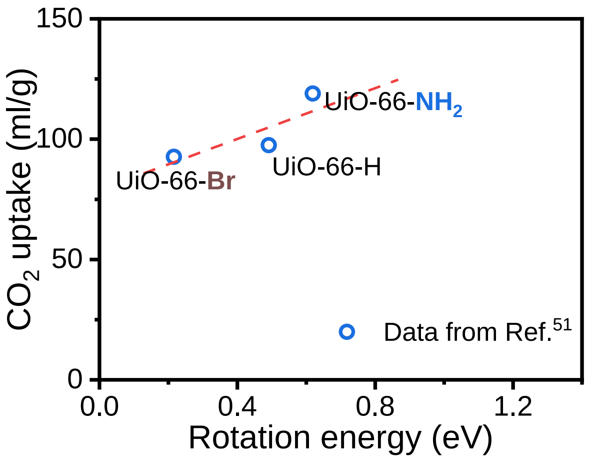

Supplementary Figure 13. Correlation between CO_2 uptake and the rigidity of UiO-66-X. The value of CO_2 uptake is obtained from Ref.[51] and the adsorption conditions are 1 bar and 0 ^{o}C.

Supplementary Table 1. Elemental composition of UiO-66-X in the selected areas.

UiO-66-X		C (wt%)	O (wt%)	Zr (wt%)	X (wt%)
UiO-66-H	EDS	26.45%	23.74%	59.35%	
	Theoretical	34.66%	30.81%	32.85%	
UiO-66-NH_2	EDS	50.52%	18.35%	27.16%	3.97%
	Theoretical	32.88%	29.22%	31.16%	4.79%
UiO-66-Cl	EDS	24.59%	19.94%	40.96%	14.51%
	Theoretical	30.82%	27.40%	29.22%	11.38%
UiO-66-Br	EDS	27.03%	15.91%	37.99%	19.07%
	Theoretical	26.97%	23.97%	25.56%	22.47%

Supplementary Table 2. Hydrogen bond between oxygen in carboxyl group and hydrogen in the functional group.

UiO-66-X	Rotation energy (eV)	$d_{H\ldots O}$ (Å)	$\theta_{X\text{-}H\ldots O}$	$-ICOHP_{H\ldots O}$ at E_F (eV)
UiO-66-CH_3	0.33	2.37	94.4°	0.04
UiO-66-NH_2	0.62	1.88	127.0°	0.51
UiO-66-OH	0.89	1.64	148.5°	1.22

Supplementary Table 3. Fitted results of FWHM against rotation energies.

Parameters	d/Å	E_0/eV	d_{atom}/Å
Fitted results	1.89±0.22	0.18±0.06	1.82±0.26

Supplementary Table 4. Rotation properties of UiO-66-X.

UiO-66-X	Rotation energy at 90° (eV)	Average rotation degree at RT (°)
UiO-66-H	0.49	33
UiO-66-CH_3	0.33	48
UiO-66-NH_2	0.62	26
UiO-66-OH	0.89	18
UiO-66-F	0.36	44
UiO-66-Cl	0.24	67
UiO-66-Br	0.22	75
UiO-66-OH-Rev	0.29	
